# miR‐34a promotes the immunosuppressive function of multiple myeloma‐associated macrophages by dampening the TLR‐9 signaling

**DOI:** 10.1002/cam4.7387

**Published:** 2024-06-12

**Authors:** Rui Zhang, Disi Zhang, Yilan Luo, Yunyan Sun, Ci Duan, Jiapeng Yang, Jia Wei, Xianshi Li, Yanqi Lu, Xun Lai

**Affiliations:** ^1^ Department of Hematology Yunnan Cancer Hospital, The Third Affiliated Hospital of Kunming Medical University, Peking University Cancer Hospital Yunnan No.519 Kunzhou Road, Xishan District Kunming Yunnan Province China; ^2^ Department of Thoracic Surgery Yunnan Cancer Hospital, The Third Affiliated Hospital of Kunming Medical University, Peking University Cancer Hospital Yunnan No.519 Kunzhou Road, Xishan District Kunming Yunnan Province China

**Keywords:** CAR‐T, macrophages, miR‐34a, multiple myeloma, TLR9

## Abstract

**Background:**

Promising outcomes have been observed in multiple myeloma (MM) with the use of immunotherapies, specifically chimeric antigen receptor T (CAR‐T) cell therapy. However, a portion of MM patients do not respond to CAR‐T therapy, and the reasons for this lack of response remain unclear. The objective of this study was to investigate the impact of miR‐34a on the immunosuppressive polarization of macrophages obtained from MM patients.

**Methods:**

The levels of miR‐34a and TLR9 (Toll‐like receptor 9) were examined in macrophages obtained from both healthy individuals and patients with MM. ELISA was employed to investigate the cytokine profiles of the macrophage samples. Co‐culture experiments were conducted to evaluate the immunomodulatory impact of MM‐associated macrophages on CAR‐T cells.

**Results:**

There was an observed suppressed activation of macrophages and CD4^+^ T lymphocytes in the blood samples of MM patients. Overexpression of miR‐34a in MM‐associated macrophages dampened the TLR9 expression and impaired the inflammatory polarization. In both the co‐culture system and an animal model, MM‐associated macrophages suppressed the activity and tumoricidal effect of CAR‐T cells in a miR‐34a‐dependent manner.

**Conclusion:**

The findings imply that targeting the macrophage miR‐34a/TLR9 axis could potentially alleviate the immunosuppression associated with CAR‐T therapy in MM patients.

## Graphical abstract

In multiple myeloma (MM)‐associated macrophages, miR‐34a is overexpressed leading to the downregulation of TLR9 signaling, which suppresses the inflammatory response and impairs the effectiveness of CAR‐T cells in tumor eradication.

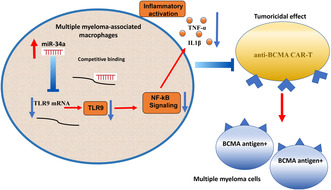

## 1 INTRODUCTION

Multiple myeloma (MM) is a prominent hematological malignancy involving plasma cells, encompassing a variety of plasma cell abnormalities like leukemia and extramedullary myeloma.^1^, ^2^ Unhealthy lifestyle habits, including chronic alcohol consumption, smoking, and dietary patterns, as well as genetic mutations in specific genes, are associated with an elevated MM risk.^3^, ^4^ Various classes of chemotherapeutic medications have been developed to target distinct cellular processes in MM treatment. Nevertheless, the emergence of drug resistance is an inevitable problem that comprises the treatment outcome.^5^, ^6^ Recent advancements in immunotherapies, such as chimeric antigen receptor T (CAR‐T) cell therapy, have offered new direction in the MM therapy.^7^, ^8^ For instance, anti‐CD19 CAR‐T therapy has demonstrated efficacy in specific MM patients due to the expression of CD19 on subsets of MM cells.^9^ Co‐administration of anti‐CD19 CAR‐T cells and stem cell therapy shows promising treatment effects for refractory multiple myeloma.^10^, ^11^ Furthermore, B‐cell maturation antigen (BCMA) is detected in nearly all MM cases, but it is barely expressed in normal tissues except plasma cells.^12^ The promising target for immunotherapy arises from the significant overexpression of BCMA in malignant plasma cells.^13^ An assessment of anti‐BCMA CAR‐T therapy in MM patients demonstrated a response rate of 72%, with 28% of subjects achieving a complete response.^14^ However, despite its efficacy, CAR‐T therapy exhibits certain limitations, including the occurrence of side effects such as cytokine‐releasing syndrome (CRS). Furthermore, a subset of patients, particularly those with relapsed and refractory disease, do not respond adequately to CAR‐T therapy.^15^, ^16^ The underlying mechanisms remains elusive.

Tumor‐associated macrophages (TAMs) are immunosuppressive cells that are functionally polarized, playing a role in supporting the progression of cancer. Recent research suggests that macrophages contribute to the growth, viability, and drug resistance of myeloma cells through both direct and indirect mechanisms.^17^, ^18^ Moreover, studies have demonstrated an expansion of TAMs in patients with MM and their involvement in suppressing the anti‐tumor activity of cytotoxic T lymphocytes (CTLs) via the PD‐1/PD‐L1 signaling pathway, thus aiding in the immune evasion of myeloma cells.^19^ Despite these findings, the precise mechanisms underlying the immunosuppressive polarization of TAMs in MM remain incompletely understood.

The miR‐34a is a well‐studied microRNA that plays crucial roles in various biological processes, including cancer development and immune regulation. In the context of cancer, miR‐34a is recognized as a tumor suppressor, and its downregulation has been observed in numerous malignancies, such as lung cancer, breast cancer, and multiple myeloma.^20^, ^21^, ^22^ Conversely, the restoration of miR‐34a expression can inhibit cancer cell proliferation, induce apoptosis, and impair metastasis.^23^ miR‐34a has also been implicated in the modulation of inflammatory responses and immune cell functions.^24^, ^25^ For instance, a growing body of evidence suggests that miR‐34a modulates T cell activation and function, suggesting its potential role in regulating adaptive immunity and anti‐cancer immunity.^26^, ^27^ Novel evidence further highlights its potential role in regulating macrophage polarization in different pathophysiological conditions.^28^, ^29^ Importantly, miR‐34a has been recognized as a potential target in MM.^30^ Elucidating the functional engagement of miR‐34a in modulating MM‐associated macrophage polarization and anti‐cancer T cell immunity can offer insights into the enhancement of immunotherapy.

Here, we aim to scrutinize the potential function of miR‐34a in dictating the immunosuppressive polarization of TAMs isolated from the MM patients and investigated the molecular mechanism. We further evaluated the impact of TAMs on anti‐BCMA CAR‐T therapy in the cell and animal model.

## 2 MATERIALS AND METHODS

### 2.1 Blood sample collection

We collected peripheral blood samples from a total of 20 individuals, consisting of 10 healthy controls and 10 patients diagnosed with MM, at Yunnan Cancer Hospital. After collection, a fraction of each blood sample was dedicated to isolating immune cells, while the remaining samples were immediately frozen using liquid nitrogen and stored at a temperature of −80°C to facilitate subsequent analyses. This research was conducted in compliance with the regulations set forth by the medical research committee of Yunnan Cancer Hospital, and informed consent was obtained from all participating individuals.

### 2.2 Macrophage and CD4 ^+^ T cell isolation

For macrophage isolation from the peripheral blood samples, Classical Monocyte Isolation Kit, human (Miltenyi Biotec, CA, USA), was used to purify classical (CD14^++^CD16^−^) monocytes based on the supplier's instructions. Dynabeads™ CD4 Positive Isolation Kit (ThermoFisher Scientific, CA, USA). was used for the isolation of CD4^+^ lymphocytes.

### 2.3 CAR‐T cell generation

The NHP/TYF system was used to generate lentiviral vectors (LV). The pTYF transducing vector was utilized to clone the CAR construct (scFv/4‐1BB/CD3ζ), which consists of a BCMA single‐chain variable fragment (scFv) linked with 4‐1BB co‐stimulatory and CD3ζ signaling region, regulated by a human EF1a promoter. Overnight stimulation of patient‐derived PBMCs was conducted using magnetic beads coated with anti‐CD3/CD28 antibodies (ThermoFisher Scientific). The subsequent day, viral infection was performed in PBMCs at a multiplicity of infection of 1:10. The efficiency of infection was determined by flow cytometry using an anti‐BCMA detection reagent.^31^ Adequate amount of CAR‐T cells were generated from pooled PBMCs for both cell and animal experiments.

### 2.4 Cell culture

The Human MM cell line MM.1S was acquired from Cobioer Biosciences, situated in Nanjing, China. The cells were maintained in McCoy's medium, consisting of 10% FBS as well as 1% penicillin/streptomycin (Hyclone in CA, USA). The cells were cultured at a temperature of 37°C with a CO_2_ concentration of 5%. Macrophages were extracted from healthy controls and MM patients and cultivated in Monocyte Culture Medium (3H Biomedical, Uppsala, Sweden). CAR‐T cells were cultivated using RPMI‐1640 medium containing 10% FBS and 5 ng/mL recombinant IL‐2 (Peprotech in CA, USA). For the co‐culture experiment involving macrophages and CAR‐T cells, a ratio of 1:1 was employed, with CAR‐T cells and macrophages plated together in a 24‐well plate for a duration of 48 h. To characterize the CAR‐T cells, they were purified using CD3^+^ magnetic beads (ThermoFisher Scientific) before any analyses were conducted. In the co‐culturing experiment between CAR‐T and MM cells, MM.1S cells were seeded with CAR‐T cells at 1:0.5 in a 24‐well plate for a duration of 48 hours prior to analysis. For macrophage stimulation, TLR9‐agonist CpG ODNs (MedChemExpress, Shanghai, China) were applied at 10 μM for 48 h.

### 2.5 Cell transfection

The synthesized MiR‐34a inhibitor and the corresponding negative control (referred to as inhibitor‐NC) were produced by RiboBio, located in Guangzhou, China. To introduce these substances into macrophages, Lipofectamine 3000 (Invitrogen, Shanghai, China) was utilized following the instructions provided by the manufacturer. For transfection purposes, a concentration of 50 nM was employed for each compound, and the subsequent functional assays were conducted 48 hours after the transfection procedure.

### 2.6 CCK‐8 proliferation assay

CAR‐T cells, with or without the presence of macrophages, were introduced into a 96‐well plate at a concentration of 4000 cells per well. The CAR‐T cells underwent activation by using anti‐CD3 (1 μg/mL) coated on the plate and soluble anti‐CD28 (1 μg/mL) for 24 h. Following this, a volume of 10 μL of CCK8 reaction solution (Solarbio, Beijing, China) was incorporated into the cell culture, and the cells were incubated for 3 h. The absorbance of light at a wavelength of 450 nm was then measured using a Synergy H1 microplate reader (Winooski, Vermont, USA) for each specific condition.

### 2.7 Lactate dehydrogenase (LDH) assay

Lactate Dehydrogenase (LDH) activity was assessed utilizing the LDH Assay kit (Beyotime, Beijing, China). The 50 μL of the cell culture supernatant and standards were combined with an equal volume (50 μL) of the LDH Reaction Mix. The mixture was then incubated for 30 minutes at 37°C. Following a gentle agitation, the signal intensity was quantified at 450 nm using a microplate reader set at 37°C.

### 2.8 Apoptosis analysis

Apoptotic events were detected using the FITC Annexin V Apoptosis Detection Kit (BD Biosciences, CA, USA) following the instructions provided by the manufacturer. In summary, a total of 1 × 10^6^ cells were stained with 5 μL of Annexin V‐FITC and 5 μL of PI, incubated for 30 min in darkness. Subsequently, the stained cells underwent centrifugation and were washed twice with the provided washing buffer. The BD FACS CantoTM II Flow Cytometer (BD Biosciences, CA, USA) was then employed to determine the percentage of apoptotic cells.

### 2.9 Dual luciferase reporter assay

To investigate the functional relationship between miR‐34a and TLR9 mRNA, the researchers utilized the PmirGLO firefly luciferase reporter. They inserted two versions of the sequence into the reporter: one with the predicted wild‐type binding sites (WT) and another with mutated binding sites (MUT). Additionally, they co‐transfected the reporter plasmid with a control plasmid containing Renilla luciferase (hRlucneo) into 293 T cells. The transfection was performed in the presence of either a miR‐24a mimic or a non‐targeting control (miR‐NC), using Lipofectamine 3000 reagent. After 48 h, the researchers measured the luciferase activity relative to the controls using the Dual‐Luciferase Reporter Assay Kit (ThermoFisher Scientific) on a luminescence reader. This allows the evaluation of the effects of miR‐34a on the luciferase activity driven by the different versions of the binding site sequences in the reporter.

### 2.10 ELISA

In this study, the levels of IL‐1β, TNF‐α, IL‐10, TGF‐β1, and IL‐2 in the samples were analyzed using corresponding commercial ELISA kits (Sigma, Shanghai, China). The assay plate was coated with specific antibodies and filled with the samples or standards for incubation. Biotin‐labeled detection antibody and streptavidin‐HRP reagent were added to label the bound materials. After washing, chemiluminescent detection reagent was used to develop the signal, and the optical densities were recorded at 450 nm.

### 2.11 qRT‐PCR analysis

The RNA extraction process was carried out using TRIzol reagent (Qiagen, Shanghai, China). The extracted RNA was then subjected to reverse transcription using the PrimeScript™ RT Reagent Kit (Takara, Dalian, China). After the reverse transcription, qPCR analysis was performed using the SYBR premix EX TAQ II kit (Takara) in the 7500 Real Time PCR System. The 2‐ΔΔCT method was used to determine the relative gene expression, with β‐actin serving as the internal control.

### 2.12 Western blot

The experiment involved the use of RIPA lysis buffer supplemented with protease inhibitor and PMSF for protein sample extraction. The samples were then subjected to a BCA assay kit (Beyotime, Beijing, China) for protein concentration measurement. The denatured protein samples were separated in 12% SDS‐PAGE and transferred onto a PVDF membrane. Primary antibodies from Abcam were used to probe the membrane. These included anti‐beta actin antibody (ab8227, 1:2000), anti‐LCK (ab3885, 1:1000), anti‐p‐LCK (ab138442, 1: 1500), anti‐ZAP70 (ab32410, 1:1500), anti‐p‐ZAP70 (ab194800, 1:1500), anti‐TLR9 (ab211012, 1:2000), anti‐IKKβ (ab124975, 1:1500), anti‐p‐IKKβ (ab194845, 1:1500), anti‐IkB‐α (ab97783, 1:1500), anti‐CD206 (ab125028, 1:1500) and anti‐iNOS (ab15323, 1:1500). After washing with TBST buffer, the membrane was incubated with a horseradish peroxidase‐conjugated secondary antibody. The protein bands were developed using an ECL chemiluminescent solution (Beyotime).

### 2.13 Animal model

Balb/c nude mice (male, 6 weeks old) were obtained from the Shanghai Laboratory Animal Center (SLAC) Co., Ltd. All the protocols were performed according to the guidelines approved by the Institutional Animal Care and Use Committee (IACUC) of Yunnan Cancer Hospital (Kunming, China). The mice were divided into five groups (*n* = 5 animals in each group): control (injected with 5 × 10^6^ MM.1S cells); CAR‐T group (injected with 5 × 10^6^ MM.1S cells and 1 × 10^6^ CAR‐T); CAR‐T + M group (injected with 5 × 10^6^ MM.1S cells, 1 × 10^6^ CAR‐T and 1 × 10^6^ macrophages); CAR‐T + M + miR‐34a inh group (injected with 5 × 10^6^ MM.1S cells, 1 × 10^6^ CAR‐T and 1 × 10^6^ macrophages, and administered with 1 mg/kg miR‐34a inhibitor every week); CAR‐T + M + CpG ODN group (injected with 5 × 10^6^ MM.1S cells, 1 × 10^6^ CAR‐T and 1 × 10^6^ macrophages, and administered with 5 mg/kg CpG ODN every week). The tumor growth was monitored every 7 days, and all the animals were euthanized on day 35, and the xenograft samples were collected for further analysis. The cell death events in the tumor tissues were examined using the TUNEL tissue assay kit (Beyotime).

### 2.14 Statistical analysis

Data were summarized as mean ± SD, and the GraphPad Prism software (GraphPad Software, NY, USA) was used to analyze the data. The student's t‐test was employed to compare the difference between two groups, and for multiple comparisons, one‐way ANOVA was utilized with a predefined statistical threshold of P < 0.05.

## 3 RESULTS

### 3.1 Suppressed activation of macrophages and CD4 ^+^ T lymphocytes in MM patients

**FIGURE 1:**
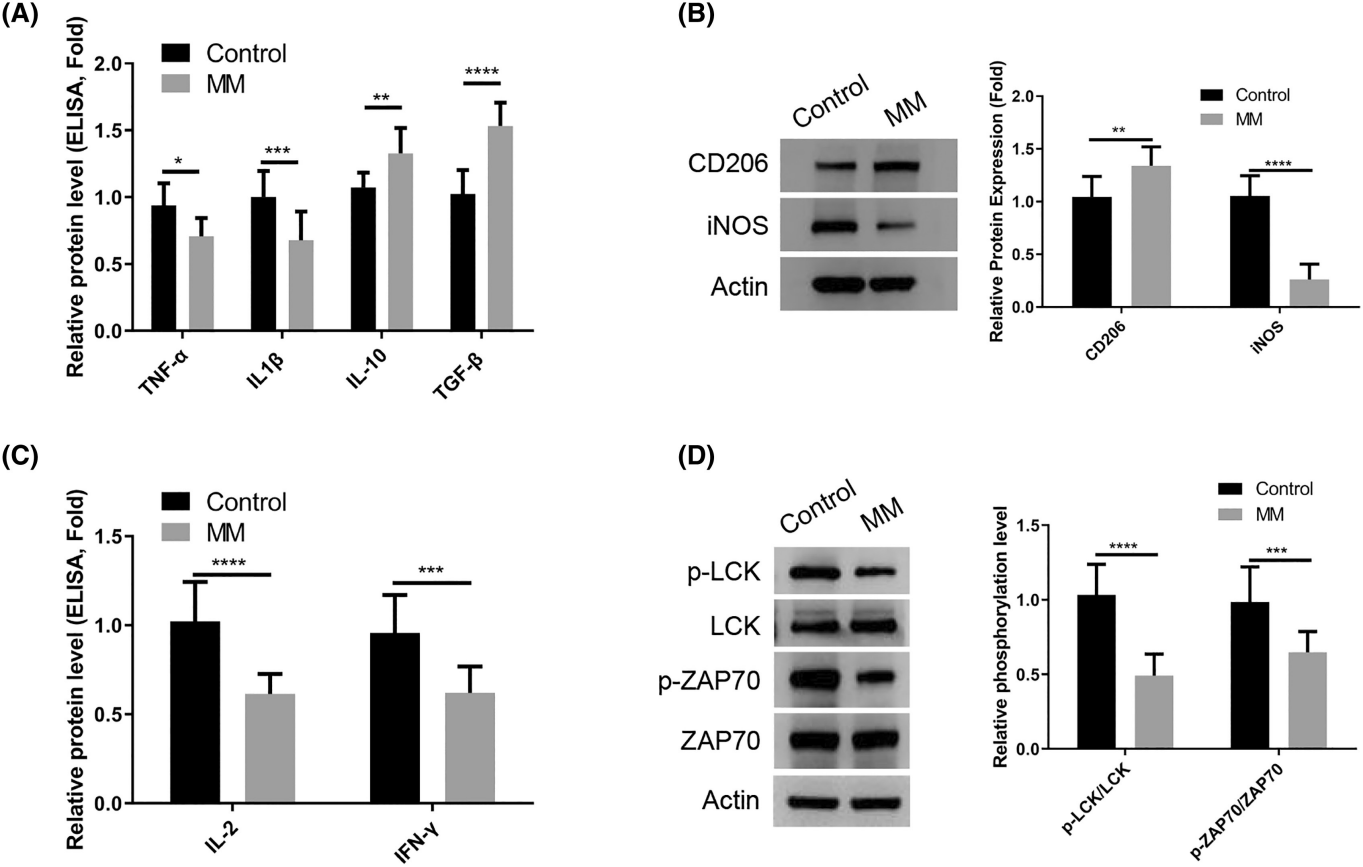
Suppressed activation of macrophages and CD4+ T lymphocytes in the blood samples of MM patients. (A). ELISA analysis of TNF‐α、IL1β (M1‐type cytokines) and IL‐10 and TGF‐β1 (M2‐type cytokines) in the macrophages from the healthy controls and MM patients. (B). Western blot analysis of M2 marker CD206 and M1 marker iNOS in the macrophages isolated from the healthy controls and MM patent samples. (C). ELISA analysis of IL‐2 and IFN‐γ in the CD4^+^ T cells isolated from the healthy controls and MM patients. (D). Western blot analysis of the relative phosphorylation levels of LCK and ZAP70 in the CD4^+^ T cells isolated from the healthy controls and MM patients. (D) Data were summarized as mean ± SD from 10 clinical samples in each group. *p < 0.05, **p < 0.01, ***p < 0.001, ****p < 0.0001.

In order to compare the activation patterns of macrophages and CD4^+^ T lymphocytes in healthy controls and patients with MM, peripheral blood samples were collected from each group for isolation of immune cells. Examination of isolated macrophages using ELISA analysis revealed a decrease in the production of M1‐type cytokines TNF‐α and IL1β in MM patients compared to healthy controls, while M2‐type cytokines IL‐10 and TGF‐β1 were found to be increased in MM patients (Figure 1A). Additionally, Western blot analysis confirmed an elevated expression of M2 marker CD206 and a reduced expression of M1 marker iNOS in macrophages isolated from MM patient samples (Figure 1B). Furthermore, isolated CD4^+^ T cells from blood samples of MM patients exhibited a decrease in the production of IL‐2 and IFN‐γ (Figure 1C). Evaluation of T cell receptor (TCR)‐mediated signaling activation by measuring relative phosphorylation levels of LCK and ZAP70 revealed a significant attenuation in CD4^+^ T cells isolated from MM patients compared to healthy controls (Figure 1D). Taken together, these findings suggest a tendency of macrophages in MM patients to display features associated with anti‐inflammatory M2 polarization, while CD4^+^ T cells show dampened TCR signaling.

### 3.2 The overexpression of miR‐34a in macrophages from MM patients suppresses TLR9 expression

**FIGURE 2:**
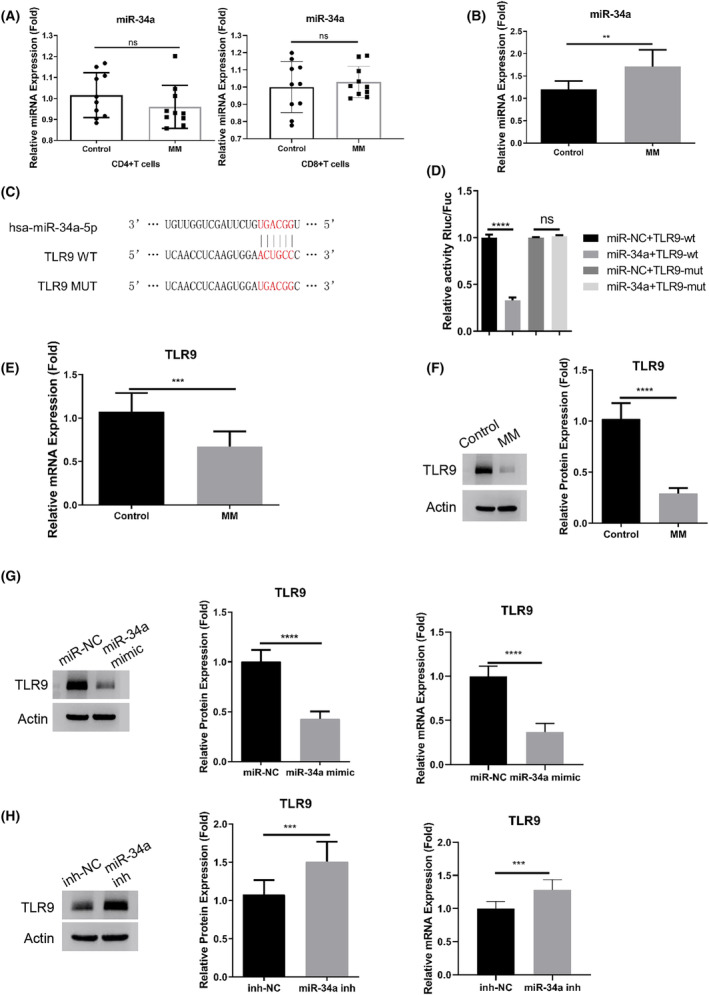
The upregulation of miR‐34a in macrophages from MM patients suppresses TLR9 expression. (A). qRT‐PCR analysis of miR‐34a in the CD4^+^ and CD8^+^ T cells isolated from the healthy controls and MM patients. (B). qRT‐PCR analysis of miR‐34a in the macrophages isolated from the healthy controls and MM patients. (C). Bioinformatics prediction of the interaction sites between using the TargetScan resources, we found that there were potential interaction sites between miR‐34a and TLR9 mRNA 3'UTR using TargetScan database. (D). Dual luciferase reporter assay using WT and MUT reporter in the presence of miR‐NC (miRNA mimic control) or miR‐34a mimic. (E). qRT‐PCR and (F). Western blot analysis of TLR9 in the macrophages isolated from the healthy controls and the MM patients. (G). Western blot and qRT‐PCR analysis of TLR9 expression the transfection of miR‐34a mimic or miR‐NC. (H). Western blot and qRT‐PCR analysis of TLR9 expression the transfection of miR‐34a inhibitor or inh‐NC (inhibitor control). Data in A, B, and E were summarized as mean ± SD from 10 clinical samples in each group. Data in D, F, G and H were summarized from 3 independent experiments. *p < 0.05, **p < 0.01, ***p < 0.001, ****p < 0.0001.

We subsequently investigated miR‐34a expression levels in macrophages, CD4^+^ Th cells, and CD8^+^ cytotoxic T cells obtained from both controls and MM patients. Macrophages isolated from MM patients displayed a significant increase in miR‐34a expression. Conversely, the expression level of miR‐34a remained unchanged in both CD4^+^ and CD8^+^ T cells when comparing healthy controls to MM patients (Figure 2A,B). Our curiosity about the impact of miR‐34a on macrophage activation prompted us to utilize bioinformatics prediction through TargetScan resources. This analysis revealed potential interaction sites between miR‐34a and TLR9 mRNA 3'UTR (Figure 2C). To further explore this interaction, we cloned wild‐type (WT) and mutated (MUT) binding sites into a luciferase reporter. Dual luciferase reporter assay demonstrated that miR‐34a mimic significantly suppressed the activity of the WT reporter, while no effect was observed in the MUT reporter (Figure 2D), indicating an interaction through the predicted binding sites. Additionally, qRT‐PCR and Western blot analysis affirmed a significant downregulation of TLR9 in macrophages isolated from MM patients (Figure 2E,F). Furthermore, transfection of miR‐34a mimic led to suppressed TLR9 expression in macrophages at both mRNA and protein levels, whereas miR‐34a inhibitor promoted its expression (Figure 2G,H). In summary, these findings suggest that miR‐34a functions as a negative regulator of TLR9 in macrophages.

### 3.3 miR‐34a dependent TLR9 downregulation mediates the inflammatory activation of macrophages

**FIGURE 3:**
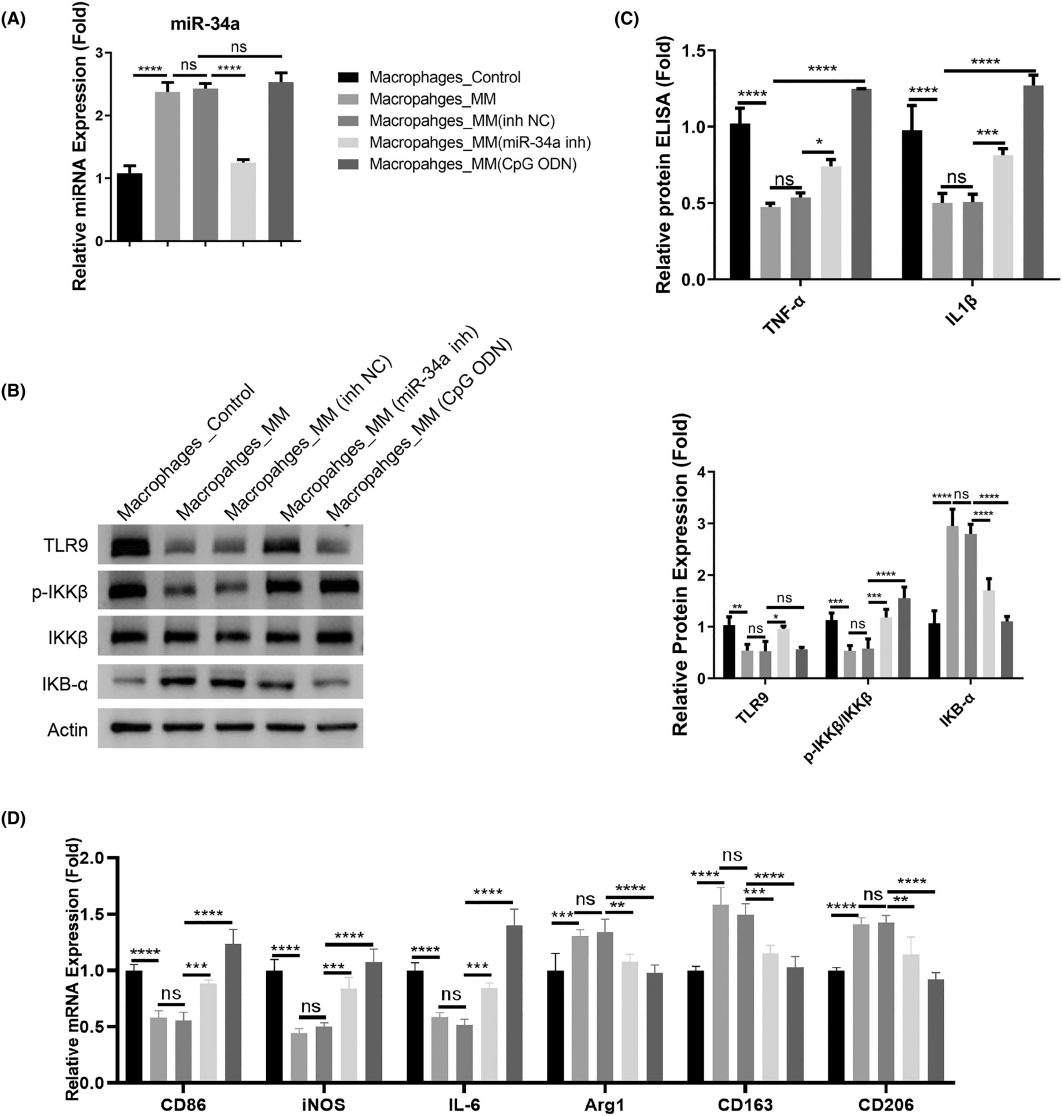
The miR‐34a dependent TLR9 downregulation mediates the inflammatory activation of macrophages. The macrophages isolated from the MM patients were transfected with miR‐NC or miR‐34a inhibitor. TLR9 agonist CpG ODN was also applied in the macrophages to examine the effect on the production of inflammatory cytokines. (A). qRT‐PCR analysis of miR‐34a expression levels. (B). Western blot analysis of TLR9, the phosphorylation levels of IKKβ and the protein levels of IkB‐α. (C). ELISA analysis of TNF‐α and IL1β in each experimental group. (D). RT‐qPCT analysis of M1 marker genes (CD86, iNOS, and IL‐6) and M2 marker genes (Arg1, CD163, and CD206). Data were summarized as mean ± SD from 3 independent experiments.*p < 0.05, **p < 0.01, ***p < 0.001, ****p < 0.0001.

To show whether miR‐34a‐dependent TLR9 downregulation controls the inflammatory phenotype in macrophages, macrophages isolated from the MM patients were subjected to the transfection of control or miR‐34a inhibitor. TLR9 agonist CpG ODN was also applied in the macrophages to detect the effect on inflammatory factors. The miR‐34a upregulation was suppressed after miR‐34a inhibitor transfection, but TLR9 agonist did not affect miR‐34a expression (Figure 3A). As expected, miR‐34a inhibition promoted TLR9 expression, and also reactivated the TLR9 downstream signaling in the macrophages from the MM patients (the phosphorylation of IKKβ and the degradation of IkB‐α). TLR9 agonist treatment showed a similar effect (Figure 3B). After the introduction of miR‐34a inhibitor and TLR9 agonist treatment, there was a significant increase of TNF‐α and IL1β in the macrophages from the MM patients (Figure 3C). Furthermore, we measured the expression of M1 marker genes (CD86, iNOS, and IL‐6) and M2 marker genes (Arg1, CD163, and CD206). Similarly, the introduction of miR‐34a inhibitor and TLR9 agonist promoted the expression of M1 markers in MM‐associated macrophages, while M2 marker genes showed downregulations after miR‐34a inhibition or TLR9 activation (Figure 3D). Thus, these data suggest that miR‐34a‐dependent TLR9 downregulation accounts for the suppressed inflammatory activation of macrophages in the MM patients.

### 3.4 Macrophages from the MM patients dampen the activation of CAR‐T in a miR‐34a dependent manner

**FIGURE 4:**
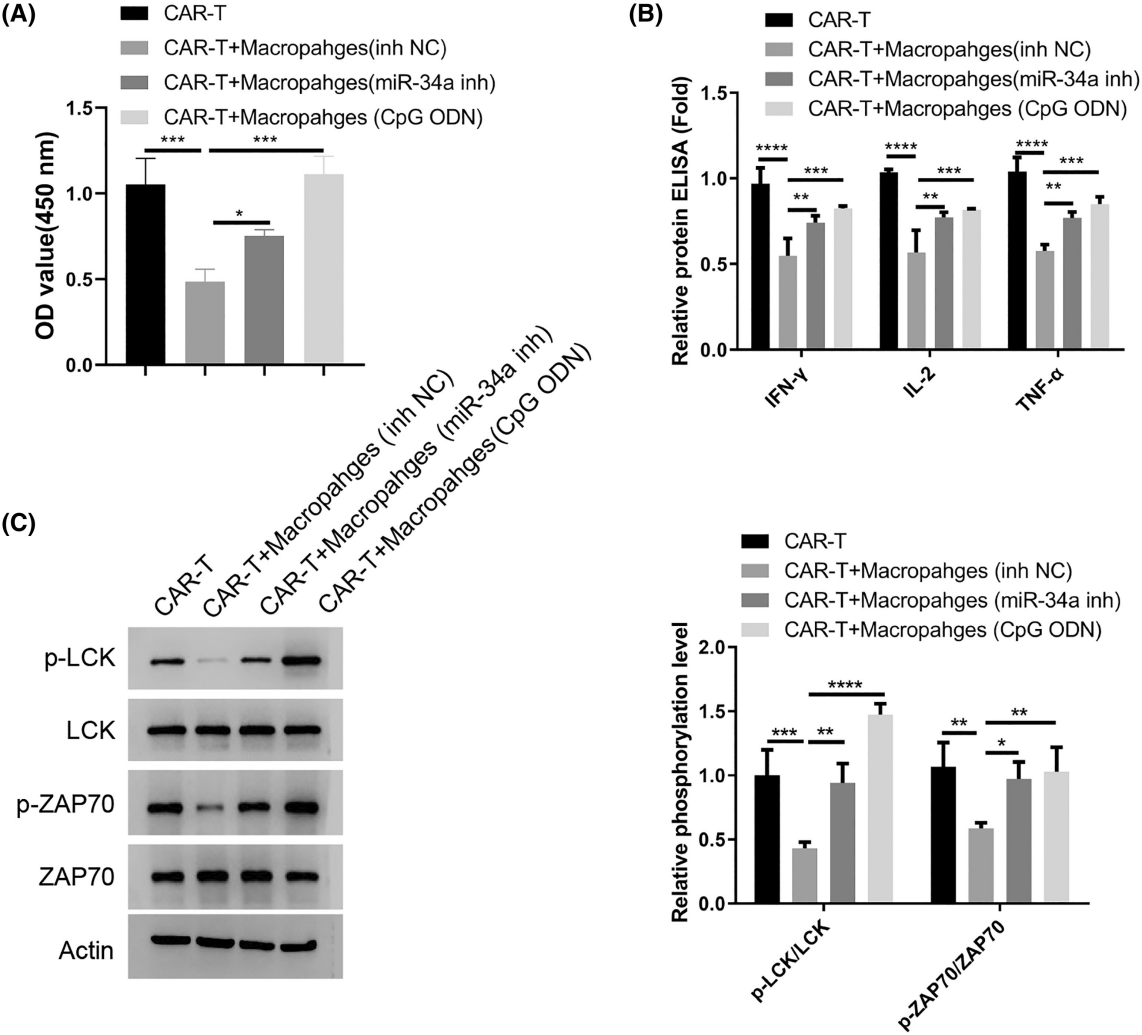
Macrophages from the MM patients dampen the activation of CAR‐T in a miR‐34a dependent manner. The macrophages from MM patients were transfected with miR‐34a inhibitor or treated with TLR9 agonist, and then introduced into co‐culture system with CAR‐T cells targeting MM cells. (A). CCK‐8 proliferation assay in the CAR‐T cells. (B). ELISA analysis of IFN‐γ、IL‐2 and TNF‐α. (C). Western blot analysis of TCR‐dependent signaling (phosphorylation of LCK and ZAP70). Data were summarized as mean ± SD from 3 independent experiments. *p < 0.05, **p < 0.01, ***p < 0.001, ****p < 0.0001.

Next, we wonder whether the state of macrophages in the MM patients impinges on the T cell activity since there was a significant suppression of TCR‐mediated signaling in the CD4^+^T cells (Figure 1D). We generated CAR‐T cells targeting MM cells and co‐cultured them with the macrophages from MM patients after miR‐34a inhibition or TLR9 agonist treatment. The macrophages transfected with miR‐NC inhibited CAR‐T cell proliferation and suppressed the synthesis of inflammatory cytokines (Figure 4A,B). With miR‐34a inhibition or TLR9 activation, the suppression on CAR‐T cells became largely abrogated. Meanwhile, the macrophages from MM patients also attenuated the activation of TCR‐dependent signaling (phosphorylation of LCK and ZAP70), while miR‐34a inhibition or TLR9 activation abolished the effect (Figure 4C). Together, these data indicate that macrophages from the MM patients dampen the activation of CAR‐T in a miR‐34a/TLR9‐dependent manner.

### 3.5 Macrophages from the MM patients suppress the tumoricidal activity of CAR‐T cells in a miR‐34a/TLR9‐dependent mechanism

**FIGURE 5:**
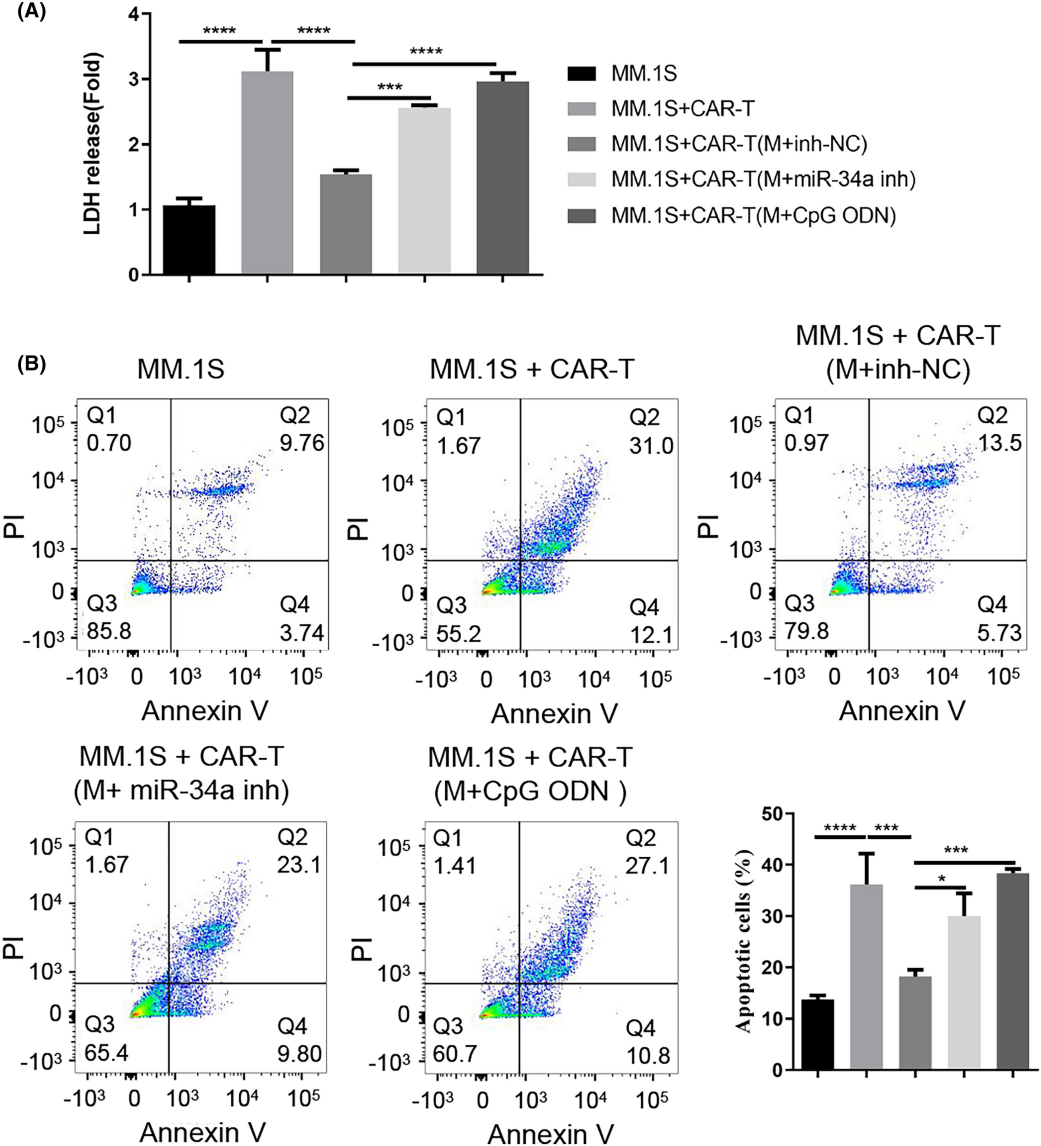
Macrophages from the MM patients suppress the cytotoxic effect of CAR‐T cells on MM cells in a miR‐34a/TLR9‐dependent manner. CAR‐T cells after the co‐culturing with different groups of macrophages (with inhibitor NC, miR‐34a inhibitor or TLR9 agonist) were plated with the MM cell line MM.1S. (A). LDH cytotoxicity assay and (B) apoptosis analysis by flow cytometry. Data were summarized as mean ± SD from 3 independent experiments. *p < 0.05, **p < 0.01, ***p < 0.001, ****p < 0.0001.

To explore the influence of macrophages on CAR‐T cell activity, CAR‐T cells after the co‐culturing with different groups of macrophages (with inhibitor NC, miR‐34a inhibitor, or TLR9 agonist) were plated with the MM cell line MM.1S. We then performed LDH cytotoxicity assay and apoptosis analysis to examine the tumoricidal activities of these CAR‐T cells after co‐culturing with macrophages. As expected, CAR‐T cells alone showed significant cytotoxic effect on MM.1S cells and induced strong apoptosis (Figure 5A,B). After the co‐culture with macrophages from MM patients, the cytotoxic and apoptosis‐inducing effects of CAR‐T cells were impaired. Nevertheless, when miR‐34a inhibitor were introduced into macrophages or macrophages were subjected to TLR9 agonist treatment, their suppressive effect on CAR‐T cells was greatly abolished (Figure 5A,B). Together, these findings suggest that macrophages from the MM patients suppress the tumoricidal activity of CAR‐T cells in a miR‐34a/TLR9‐dependent manner.

### 3.6 Macrophages from the MM patients suppress the anti‐tumorigenic activity of CAR‐T cells in the mouse model

**FIGURE 6:**
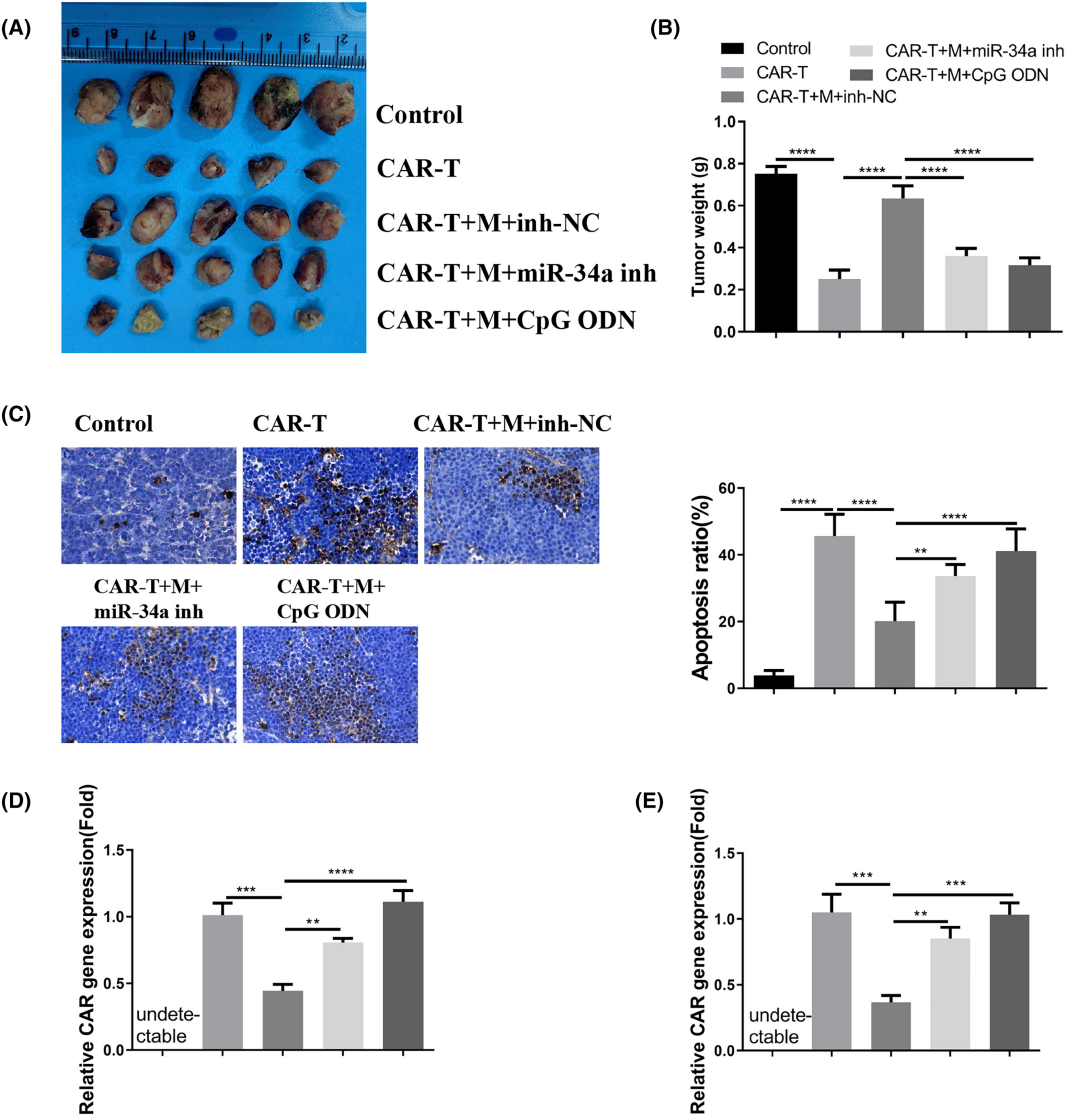
Macrophages from the MM patients suppress the anti‐tumorigenic effect of CAR‐T cells on MM cells. Nude mice were injected with MM.1S cells (control group), MM.1S/CAR‐T cells (CAR‐T group), MM.1S/CAR‐T cells and macrophages (CAR‐T + M group), MM.1S/CAR‐T cells, macrophages and miR‐34a inhibitor (CAR‐T + M + miR‐34a inh group) or MM.1S/CAR‐T cells, macrophages and TLR9 agonist (CAR‐T + M + CpG ODN group). (A). The images of xenograft tumors in each experimental group. (B). The summary of xenograft tumor weight in each experimental group. (C). TUNEL staining in the xenograft tumor tissues. The detection of CAR gene in the blood samples (E) and tumor tissues (F). Data in B, C, D, and E were summarized as mean ± SD from 5 xenograft samples in each experimental group. *p < 0.05, **p < 0.01, ***p < 0.001, ****p < 0.0001.

In order to further demonstrate the impact of macrophages derived from patients with MM on the in vivo anti‐cancer efficacy of CAR‐T cells, nude mice were administered with MM.1S cells (control group), MM.1S/CAR‐T cells (CAR‐T group), MM.1S/CAR‐T cells along with macrophages (CAR‐T + M group), MM.1S/CAR‐T cells, macrophages, and miR‐34a inhibitor (CAR‐T + M + miR‐34a inh group), or MM.1S/CAR‐T cells, macrophages, and TLR9 agonist (CAR‐T + M + CpG ODN group). The concurrent administration of CAR‐T cells significantly impeded xenograft tumor growth, while the presence of macrophages from MM patients diminished the inhibitory effect. Treatment with miR‐34a inhibitor or TLR9 agonist mitigated the suppressive influence of macrophages on CAR‐T cells (Figure 6A,B). TUNEL staining revealed that the administration of CAR‐T cells induced cell death in xenograft tumor tissues, whereas macrophages countered the anti‐tumor effect of CAR‐T cells. The administration of miR‐34a or TLR9 agonist effectively reversed the impact of macrophages (Figure 6C). Detection of the CAR gene in blood samples and tumor tissues indicated that macrophages derived from MM patients impeded the proliferation of CAR‐T cells, and treatment with miR‐34a or TLR9 agonist mitigated this effect of macrophages (Figure 6D,E). Altogether, these results suggest that macrophages obtained from MM patients repress the anticancer activity of CAR‐T cells in a miR‐34a/TLR9‐dependent manner.

## 4 DISCUSSION

In this study, we reported the suppressed activation of macrophages and CD4^+^ T lymphocytes in the blood samples of MM patients. High level of miR‐34a expression in MM‐associated macrophages dampened TLR9 expression and impaired the inflammatory polarization. Macrophages from the MM patients suppress the activation and tumoricidal activity of CAR‐T in a miR‐34a‐dependent mechanism. Together, our results suggest that targeting macrophage miR‐34a/TLR9 axis could potentially mitigate the immunosuppression on CAR‐T therapy in MM treatment.

There is compelling evidence to suggest that tumor‐associated macrophages (TAMs) contribute to the immunosuppressive function observed in both solid tumors and hematological malignancies, primarily due to their specialized polarization.^32^ In the case of patients with MM, the polarization state of macrophages has been proposed as an independent prognostic factor with the potential to be incorporated into the assessment and treatment strategies for MM.^33^ It is worth noting that MM‐associated macrophages display pro‐myeloma properties, and the abundance of these cells is inversely correlated with patient survival.^34^ Within the microenvironment of myeloma, macrophages have the ability to shield myeloma cells from undergoing apoptosis induced by chemotherapy drugs.^34^ Furthermore, in Immune Checkpoint Blockade (ICB) therapy, macrophages play a role in limiting the infiltration of CD8^+^ cytotoxic T cells into tumors, thereby constraining the efficacy of anti‐PD‐1 treatment.^35^ Moreover, tumor‐associated macrophages also contribute to immune evasion during cancer immunotherapy.^36^ However, the impact of macrophages on the tumoricidal activity of CAR‐T cells remains largely unknown.

We demonstrated that the proliferation and activation of CAR‐T was dampened by MM‐associated macrophages through the attenuation of TCR‐dependent signaling. While CARs have been developed to mirror TCR signaling, it has been observed that canonical TCRs exhibit a greater sensitivity to antigen stimulation.^37^ Optimal proliferation and activation of CAR‐T cells necessitate both CAR and TCR signaling.^38^ Additionally, various cytokines have the potential to fine‐tune the activity of CAR‐T cells.^39^ A previous investigation revealed that macrophage‐derived IL‐10 inhibits CD8^+^ T Cell Responses to chemotherapy.^40^ Furthermore, we observed increased production of anti‐inflammatory cytokines, such as IL‐10 and TGF‐β1, from MM‐associated macrophages, while levels of pro‐inflammatory cytokines were suppressed. Consequently, the anti‐inflammatory properties exhibited by MM‐associated macrophages may diminish the tumoricidal effects of CAR‐T cells against MM cells.

Toll‐like receptor (TLRs)‐mediated signaling plays a crucial role in activating nuclear factor‐kappaB (NF‐κB), a transcription factor responsible for the inflammatory function of macrophages.^41^, ^42^ Our experimental results indicate a decrease in TLR9 expression and attenuated NF‐κB signaling (IKKβ phosphorylation and IkB‐α degradation) in macrophages associated with MM. By using a TLR9 agonist called CpG ODN, we were able to reactivate the NF‐κB signaling pathway and enhance the production of inflammatory cytokines such as TNF‐α and IL1β in MM‐associated macrophages. Furthermore, the TLR9 agonist reversed the suppressive impact that MM‐associated macrophages had on CAR‐T cells. These observations lead us to propose that the weakened TLR9 signaling might contribute to the immunosuppressive function of MM‐associated macrophages. Our findings also support a previously reported link between TLR9, NF‐kB signaling, and the regulation of NLRP3 inflammasome activation.^43^

Accumulating evidence pinpoints miR‐34a as a therapeutic target in MM.^30^ Novel evidence also highlights its potential role in regulating macrophage polarization in different pathophysiological conditions.^28^, ^29^ For instance, miR‐34a is implicated in macrophage functional differentiation through targeting liver X receptor α in atherosclerosis.^28^ We further showed the overexpression of miR‐34a in MM‐associated macrophages, which accounts for the downregulation of TLR9 signaling. Inhibiting miR‐34a promoted TLR9 expression and curtailed the inhibitory effect of MM‐associated macrophages on CAR‐T cells both in vitro and in vivo. These findings are in agreement with the report that miR‐34a suppresses the inflammatory polarization of M1 macrophages in cultured murine macrophages and in the mouse model of osteolysis.^44^, ^45^

## 5 CONCLUSIONS

In summary, we reported miR‐34a overexpression and an impairment of TLR9 signaling in MM‐associated macrophages. miR‐34a was found to be a negative regulator of TLR9, which dampens the inflammatory polarization. MM‐associated macrophages suppressed the activation of CAR‐T cells as well as the tumoricidal activity on MM cells. Inhibiting miR‐34a or re‐activating TLR9 signaling in MM‐associated macrophages diminished the immunosuppressive effect. These findings imply that targeting miR‐34a in MM‐associated macrophages may serve as a strategy to boost the tumoricidal outcome of CAR‐T immunotherapy in MM treatment.

## AUTHOR CONTRIBUTIONS

**Rui Zhang:** Conceptualization (equal); data curation (equal); formal analysis (equal); funding acquisition (equal); methodology (equal); writing – original draft (equal). **Disi Zhang:** Conceptualization (equal); data curation (equal); formal analysis (equal); resources (equal); writing – original draft (equal). **Yilan Luo:** Conceptualization (equal); data curation (equal); formal analysis (equal); writing – original draft (equal). **Yunyan Sun:** Data curation (equal); formal analysis (equal); methodology (equal). **Ci Duan:** Conceptualization (equal); formal analysis (equal); software (equal). **Jiapeng Yang:** Data curation (equal); investigation (equal); resources (equal). **Jia Wei:** Formal analysis (equal); resources (equal); visualization (equal). **Xianshi Li:** Formal analysis (equal); methodology (equal); resources (equal). **Yanqi Lu:** Methodology (equal); resources (equal). **Xun Lai:** Funding acquisition (equal); methodology (equal); resources (equal); supervision (equal); writing – review and editing (equal).

## FUNDING INFORMATION

This work was supported by 1. Qiu Lu Gui Expert Workstation in Yunnan Province (Project No. 202105AF150051); 2. National Natural Science Foundation of China (Received No. 8216010239);3. Kunming Joint Special Project (Received No. 202001AC070617); 4. Kunming Joint Special Project (Received No. 202001AC070202); 5. Yunnan Fundamental Research Projects (grant NO.202101AT070199); 6. The Joint Project of Kunming Medical University and Science and Technology Agency (grant NO.202101AY070001‐049).

## CONFLICT OF INTEREST STATEMENT

All the authors declare no conflict of interest.

## ETHICS STATEMENT

All animal protocols were performed according to the guidelines approved by the Institutional Animal Care and Use Committee (IACUC) of Yunnan Cancer Hospital (Kunming, China). The research gained the approval from the medical research committee of Yunnan Cancer Hospital.

## PATIENT CONSENT STATEMENT

All the recruited subjected provided signed form of informed consent.

## Data Availability

The corresponding author can be contacted via email to obtain the data generated in this research study, upon a reasonable request.
